# Metabolomic fingerprinting of soft tissues uncovers taxonomic, physiological, and ecological aspects of river fishes

**DOI:** 10.1007/s10695-026-01638-8

**Published:** 2026-01-22

**Authors:** Benjamin Marie, Pierre Foucault, Sébastien Duperron, Catherine Quiblier

**Affiliations:** 1 UMR 7245 CNRS-MNHN. Molécules de Communication Et Adaptation Des Microorganismes, Paris, France; 2 Paris Cité University, Paris, France

**Keywords:** Freshwater fish, Soft tissues, Untargeted metabolomics, Aquatic environment, Ecological stress

## Abstract

Recent advances in molecular phenotyping have driven the rapid growth of untargeted, multi-dimensional approaches such as epigenomics, transcriptomics, proteomics, and metabolomics. When applied to ecology, these high-throughput omics tools offer powerful new molecular trait descriptors for investigating biological and environmental processes. Using UHPLC-HRMS/MS, we analyzed metabolome variations in gut, liver, and muscle tissues of chubs and gudgeons collected in summer 2019 from French rivers affected by benthic cyanobacterial blooms. Tissue-specific metabolomic profiles were evident, with muscle metabolomes showing the most distinct species differentiation. The different tissue metabolomes of both fish species also varied by sampling location, indicating local environmental influences. Notably, fish from the Vienne site exhibited molecular signatures of metabolic stress, including elevated oxidized glutathione and bile acids, and decreased purines, amino acids, peptides, and lipids—potentially linked to anatoxin-a-producing cyanobacterial mats. These findings underscore the potential of environmental metabolomics as a sensitive tool for assessing ecological stress and support its integration into routine environmental bio-indicator programs.

**Supplementary Information:**

The online version contains supplementary material available at 10.1007/s10695-026-01638-8.

## Highlights

- LC-HRMS-based metabolomics on guts, livers, and muscles of fish from the Loire, Vienne, and Cher rivers.
- Metabolomes of both chubs and gudgeons exhibit a similar relation to local environmental conditions.
- Metabolites discriminating the different metabotypes were identified and suggest a stress response in fish from river Vienne.
- Muscle metabolomes exhibit an even more discriminant signature than liver or gut.

**Graphical Abstract:**

**Supplementary Information:**

The online version contains supplementary material available at 10.1007/s10695-026-01638-8.

## Graphical abstract

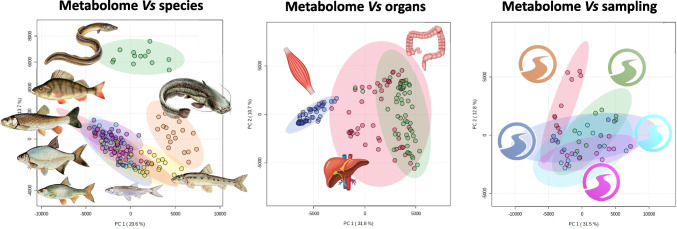

## Introduction

Biological indicators are widely employed in natural ecosystems to better understand the complex relationships between environmental conditions and organism health (Lorenz 2003). Both biotic and abiotic environmental factors are believed to shape the physiological state of bioindicator organisms. Accordingly, the measurement of specific biological markers can offer more precise insights into global environmental health (Adams and Greeley 2000).

To assess the impacts of multiple stressors on aquatic biota, novel and sensitive biological monitoring tools are required. Among these, next-generation omics technologies—particularly metabolomics—show great promise in distinguishing between reference and impacted environments and in identifying the effects of individual stressors (Marie 2020). The metabolome, defined as the complete set of primary and secondary metabolites within a biological system (e.g., an organism, organ, or microbial community), represents the final downstream output of systems biology. As such, it provides a detailed fingerprint of an organism’s molecular phenotype in response to its immediate environment (Goode et al. 2020). Metabolomics has proven valuable in advancing our understanding of growth, development, disease diagnosis, and biomarker discovery in aquatic species under controlled laboratory conditions (Bundy et al. 2009). However, its application as a tool for environmental bioindication in natural aquatic ecosystems remains rare (Sardans et al. 2011; Hani et al. 2021; Tsentalovich et al. 2022).

Environmental metabolomics seeks to identify molecular signatures by detecting early biochemical responses to environmental stressors in sentinel species (Jones et al. 2008; Lankadurai et al., 2013). Despite its potential, its broader adoption in environmental monitoring has been hampered by the inherent complexity and variability of biological systems across organs, individuals, and species (Islam et al. 2021). Thus, careful selection of biological material—considering organism type, developmental stage, tissue type, and sampling location—is crucial for robust study design, accurate data interpretation, and reproducibility (Morrison et al. 2007).

Using relatively large-bodied organisms like fish offers advantages in metabolomics-based monitoring due to their size, which allows for the analysis of multiple tissue types from individual specimens. Most fish-based environmental metabolomics studies have focused on single organs or whole-body homogenates to obtain sufficient material for analysis (Ekman et al. 2015; Lebeau-Roche et al. 2021). Recently, technological advances have enabled miniaturized approaches that require only a few milligrams of tissue. Using highly sensitive techniques such as ultra-high-performance liquid chromatography coupled with high-resolution mass spectrometry (UHPLC-HRMS), researchers can now analyze the metabolomes of small fish organs and invertebrates (Colas et al. 2021; Lance et al. 2023; Marie et al. 2023). While no single metabolomic snapshot can capture the entirety of an organism’s metabolic profile, UHPLC-HRMS provides excellent analytical capabilities, including high throughput, sensitivity, dynamic range, and molecular coverage—opening new possibilities for molecular phenotyping in environmental science (Beyoglu and Idle 2020).

Environmental stressors are known to induce distinct metabolic alterations in various fish organs. For instance, the gastrointestinal tract—being a primary route for pollutant entry—can reveal health-related metabolic and microbial shifts (Gallet et al. 2023). The liver, a central site of detoxification and metabolism, often exhibits strong responses to chemical stressors (Colas et al. 2020). Pilot metabolomic studies on the livers of fish collected from peri-urban lakes near Paris revealed potential metabolite changes associated with cyanobacterial exposure (Sotton et al. 2019; Marie and Gallet 2022). Meanwhile, muscle tissue, which is involved in locomotion and energy storage, may provide additional insights into the organism’s energetic and physiological status (Kim and Kim 2020).

Laboratory studies have demonstrated that metabolomic responses to stressors can be organ-specific, reflecting the unique physiological roles of different tissues (Li et al. 2012; Izral et al., 2021). However, important questions remain: How do endogenous factors like fish species or organ type influence metabolomic variability? Are responses consistent across tissues? Do similar stressors induce similar patterns of response between different fish species? Addressing these questions is essential to harness the full potential of metabolomics in environmental biomonitoring.

Based on these observations, we hypothesize that applying UHPLC-HRMS-based metabolomics to multiple organs of fish—each with distinct physiological functions—can enhance environmental diagnostics. In this study, we explore the metabolomic responses of gut, liver, and muscle tissues across two distinct fish species collected during the summer of 2019 from French rivers affected by benthic cyanobacterial proliferations. Our goal is to assess the sensitivity and organ specificity of metabolomic responses to environmental pressures, thereby advancing the application of metabolomics in aquatic environmental assessment.

## Materials and methods

### Chemicals and reagents

LC–MS grade acetonitrile, methanol, and formic acid were obtained from Carlo Erba (Val-de-Rueil, France). A standard solution of sodium formate (purchased from Sigma-Aldrich) was freshly prepared using Ultra-pure MilliQ® water (Guyancourt, France).

### Fish and biofilm sampling

**Fig. 1:**
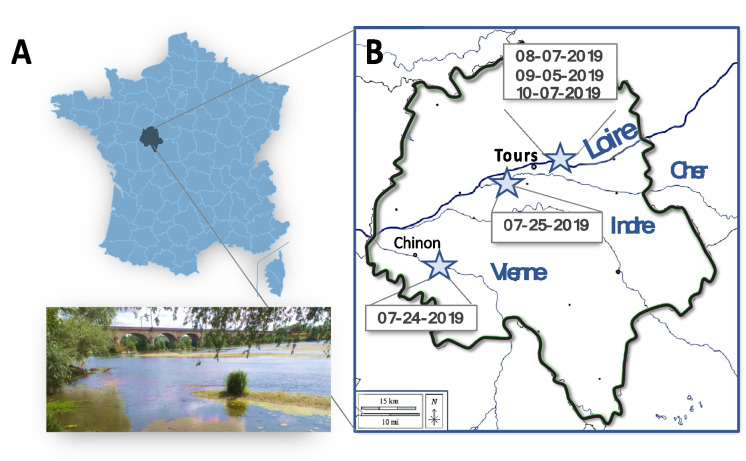
Location of the Indre et Loire French department (**A**); fish sampling sites on the Loire, Cher and Vienne rivers marked by pale blue stars (**B**); and picture of the Loire sampling area taken from the river’s banks (**C**)

Field sampling campaigns were conducted during the middle or late summer of 2019, when water levels were low, making it the most appropriate time to observe the effects of cyanobacterial mat proliferation on fishes. Five different sampling campaigns were carried out on the Loire River basin on three different rivers, with one site being sampled per river (Fig. 1). One sampling campaign took place on the Cher River on July 25, 2019, and another on the Vienne River just upstream of the city of Chinon on July 24, 2019. Monthly sampling campaigns were conducted in the Loire River, just upstream of the city of Tours, on August 7, September 5, and October 7, 2019. Fish were caught and handled in accordance with European laws. The sites were sampled using an electric fishing device (Martin Pêcheur®, Dream Electronique, France) to capture live fish, which were then promptly sacrificed and frozen. The investigation of the fish guild revealed that young chubs (*Squalius cephalus*) and gudgeons (*Gobio gobio*) were present in all sites (Table 1). These species were then selected for subsequent metabolite analyses using UHPLC-HRMS-based untargeted metabolomics.

**Table 1:** List of sample location, fish collected and investigated in this study, together with corresponding body length and number of specimens. Fish size is indicated as mean standard deviation

	Sampling	Date	Coordinates	Chub	Gudgeon	Total
				*Squalius cephalus*	*Gobio gobio*	
1	Vienne	07/24/2019	47°13’62’’ N 03°63’26’’ E	11.4 1.6 cm (n=5)	10.2 1.0 cm (n=5)	10
2	Loire_A	08/07/2019	47°39’41’’ N 08°19’31’’ E	15.6 1.7 cm (n=5)	7.8 0.6 cm (n=5)	10
3	Loire_S	09/05/2019	47°39’41’’ N 08°19’31’’ E	13.3 2.2 cm (n=5)	10.0 0.8 cm (n=5)	10
4	Loire_O	10/07/2021	47°39’41’’ N 08°19’31’’ E	17.6 4.3 cm (n=5)	11.0 1.3 cm (n=5)	10
5	Cher	07/25/2019	47°36’36’’ N 06°01’14’’ E	10.9 1.5 cm (n=5)	10.0 0.8 cm (n=4)	9

Additionally, significant developments of dark greenish microbial biofilms, likely corresponding to cyanobacterial proliferation, were observed on the riverbeds in the sampled areas. These biofilms were homogenized and frozen for further analysis in triplicates for microbial and cyanotoxin investigations, as previously described (Duperron et al. 2023; Colas et al 2020). Results of the investigations are summarized in the Supplementary Figure S1.

### Fish metabolite extraction

Fish dissection for tissue sampling (gut, liver, and white muscle) was further performed in the laboratory prior to metabolite extraction. Fish gut, liver, and muscle were thus collected and directly weighted. Then, samples were suspended in a cold extraction solvent consisting of 75–25% methanol–water, at a ratio of 1 mL per 100 mg of fresh tissues, on ice (Colas et al. 2020). A mechanical homogenization (GLH850 OMNI; 25 000 r. min^−1^; 30 s) was first carried out, then samples were sonicated (Sonics Vibra-Cell VCX 13; 60% amplitude; 30 s, × 3) and centrifuged (10 min; 4 °C; 15,300 g). The supernatant containing metabolite extracts was collected and stored at − 20 °C for subsequent metabolomic investigations using mass spectrometry.

### Untargeted metabolomic analyses by UHLC-HRMS

The metabolite composition of fish livers, guts, and muscles of chubs and gudgeons (*n* = 49) was analyzed using UHPLC (ELUTE, Bruker) coupled with a HRMS (ESI-Qq-TOF Compact, Bruker). In each sample, 2 µL was injected, and molecule separation was carried out using a Polar Advance II 2.5 pore C_18_ (Thermo Fisher Scientific, Waltham, MA, USA) chromatographic column at a flow rate of 300 μL.min^−1^ under a linear gradient of acetonitrile acidified with 0.1% formic acid (from 5 to 90% in 15 min). The electrospray ionization (ESI) system was calibrated with a capillary temperature set to 200 °C, the source voltage at 3.5 kV, and the gas flowrates at 8 L.min^−1^. Ions were then analyzed at a 2 Hz frequency acquisition rate in the range of 50–1500 m*/z* simple MS positive mode. This dataset was used for metabolite fingerprinting.

Additionally, a second analysis was conducted using collision ion dissociation (CID) on autoMS/MS in positive modes with information-dependent acquisition (IDA) on the gut, the liver, and the muscles of 6 randomly selected chubs and gudgeons. Ions with top intensities (> 5000 counts in single MS (MS1)) were thus individually selected by the quadrupole within a 10-Da window and fragmented in a collision cell (MS2) with a selective exclusion window of 30 s (except for ions showing a count-intensity increase greater than 3 times) with selective ion collision set based on ion intensity and mass (10–50 eV ramp; 50/50% time window; 2–8 Hz adapted according to ion count intensity), in consecutive cycle times of 2.5 s. The resulting ions from the fragmentation of their respective parent ions were then transferred and detected. This latter dataset was further used for metabolite annotation.

### Feature extraction

MS data were processed using MetaboScape 4.0 software (Bruker, Bremen, Germany) for recalibration of each sample analysis (< 1 ppm, according to sodium formate internal standard), peak detection, and selection of ions whose intensity was greater than 5000 counts in at least 10% of the set of samples with a minimal RT-correlation coefficient set at 0.7. Furthermore, different states of charge (1 +, 2 +, and 3 +) and classical adducts were grouped together, and the “area-under-the-peak” was determined to generate a unique global data matrix containing semi-quantification results for each metabolite in all analyzed samples’ peaks for each analyte (characterized by the respective mean mass of its neutral form and its corresponding retention time). Data QC and blank samples (injected every 4–5 samples) were examined to ensure the reproducibility and robustness of the whole data series. This step allowed us to extract features that are common among the samples in the analyzed series. We performed two distinct dataset extractions corresponding to (1) the metabolomes of the chubs and the gudgeons considering the three tissues separately and (2) the metabolomes of the chubs and the gudgeons considering all three tissues together. This data treatment strategy allowed us to maximize the extracted feature numbers for the different comparisons further performed between species and/or between tissues.

### Molecular networking and metabolite annotation

The files containing all the MS2 fragmentation information for each ion analyzed were exported in MGF format using MetaboScape 4.0 (Bruker, Bremen, Germany) software before being used for the generation of the molecular network of structural similarity using the GNPS algorithm (Wang et al. 2016) with the MetGem software (version 1.3.6) (Olivon et al. 2018) using GNPS, NIH, MS-DIAL, and EMBL public spectral databases. The annotation propagation principle was then applied to the metabolites neighboring those annotated analytes within the different molecular clusters (da Silva et al. 2015).

### Statistical analyses

For the different metabolome datasets, the MetaboAnalyst 5.0 platform (Pang et al. 2021) was used to perform data matrix normalization of metabolomic data according to *Pareto* and mean-centered, divided by the standard deviation for the different metabolite matrices. The metabolite contents were investigated and visualized on a heatmap with hierarchical classification, principal component analysis (PCA), partial least squares differential analysis (PLS-DA), one-way and two-way ANOVA with false discovery rate (FDR), and boxplots using MetaboAnalyst 5.0. Among-group variance levels were compared using PERMANOVA (999 permutations) with the Vegan package in R software (R Core Team 2024).

## Results

Global metabolomic profiling of chub and gudgeon tissues (gut, liver, and muscle) collected from five French rivers (*n* = 4–5 per site) was performed using UHPLC-MS. Two metabolomic datasets were generated.

Dataset 1 included 1355 analytes common to all three tissues (Supplementary Table S1). PERMANOVA, PCA, and heatmap analyses revealed significant differences in metabolomes across tissue types, species, and sampling locations (Fig. 1). Muscle metabolomes showed the most distinct patterns, while gut and liver metabolomes clustered more closely but exhibited stronger species-specific differences. This highlights tissue-specific molecular signatures, particularly for muscle.

**Fig. 2:**
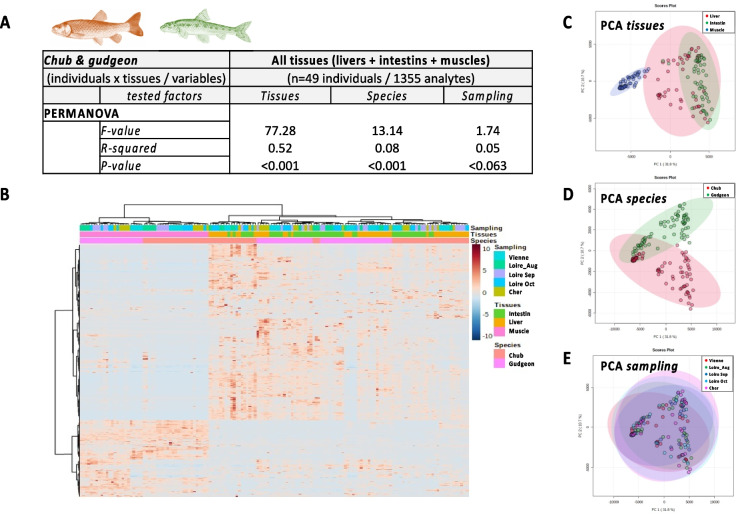
PERMANOVA analysis (**A**), heatmap with hierarchical classification (**B**), and individual plot of PCA (**C**–**E**) of metabolomes from 49 chubs and gudgeons collected in the Vienne, Cher, and Loire rivers during the 2019’s summer considering all tissues together regarding their respective tissues (**C**), species (**D**), and sampling locations (**E**)

Dataset 2 focused on tissue-specific analyses of chub and gudgeon metabolomes, comprising a greater number of analytes for each tissue (2176 in gut, 2718 in liver, 1250 in muscle; Supplementary Tables S2–S4). PERMANOVA and PCA again confirmed significant species-based differences, further supported by PLS-DA, which emphasized both species-specific patterns and clear distinctions between fish from the Vienne River and those from other rivers (Fig. 2, Table 2; Supplementary Figures S1–S2). Interestingly, samples collected one month apart in the Loire River showed minimal intra-river variability.

**Table 2:** Results of PERMANOVA analyses applied on the MS peak list of the gut, liver, and muscle list of 2176, 2718, and 1250 analytes of chubs and gudgeons according to the *species* and the *sampling* location factors

Chub and gudgeon	Gut		Liver		Muscle	
(Individuals/variables)	(*n* = 49/2176 analytes)		(*n* = 49/2718 analytes)		(*n* = 49/1250 analytes)	
*Tested factors*	*Species*	*Sampling*	*Species*	*Sampling*	*Species*	*Sampling*
**Permanova**						
*F*-value	13.24	2.06	16.16	2.31	21.16	4.14
*R*-squared	0.22	0.16	0.26	0.17	0.31	0.27
*P*-value	< 0.001	< 0.002	< 0.001	< 0.001	< 0.001	< 0.001

Molecular networking using GNPS and MetGem on 1895 MS/MS spectra (from all tissues) clustered structurally related analytes based on MS/MS fragmentation similarity (cosine score ≥ 0.65; Supplementary Figure S3). Annotation via public spectral databases and cluster propagation enabled identification of diverse metabolite classes, including nucleic acids, bile acids, amino acids and derivatives, peptides, saccharides, and lipids (e.g., LPCs, PEs, and carnitines) (Supplementary Table S5).

**Fig. 3:**
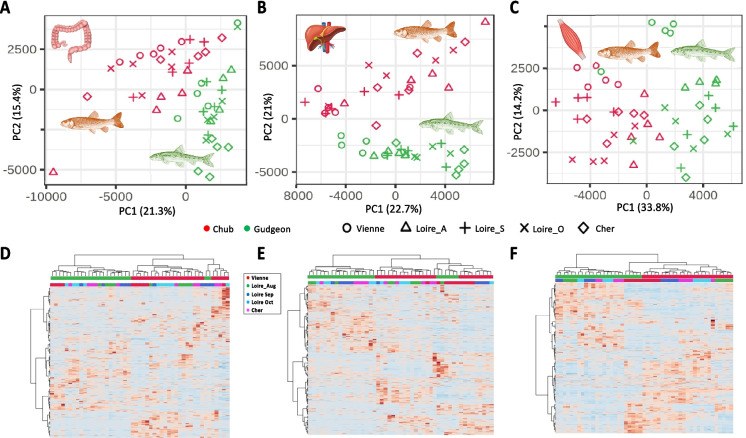
Individual plot of PCA (**A**–**C**) and heatmap with hierarchical classification (**D**–**F**) of LC–MS metabolomes from guts (**A** and **D**), livers (**B** and **E**), and muscles (**C** and **F**) of the 49 specimens of the chubs and the gudgeon collected in the Vienne, Cher, and Loire rivers during the 2019’s summer

Annotated and unannotated metabolites were subjected to ANOVA with FDR correction to assess species- and site-specific variation (*p* < 0.001; Supplementary Tables S6–S8). All tissues displayed significant metabolite abundance shifts influenced by both species and location. Notably, fish from the Vienne River exhibited consistently distinct metabolomic profiles compared to other sites, with the Cher River showing an opposite trend (Fig. 3).

**Fig. 4:**
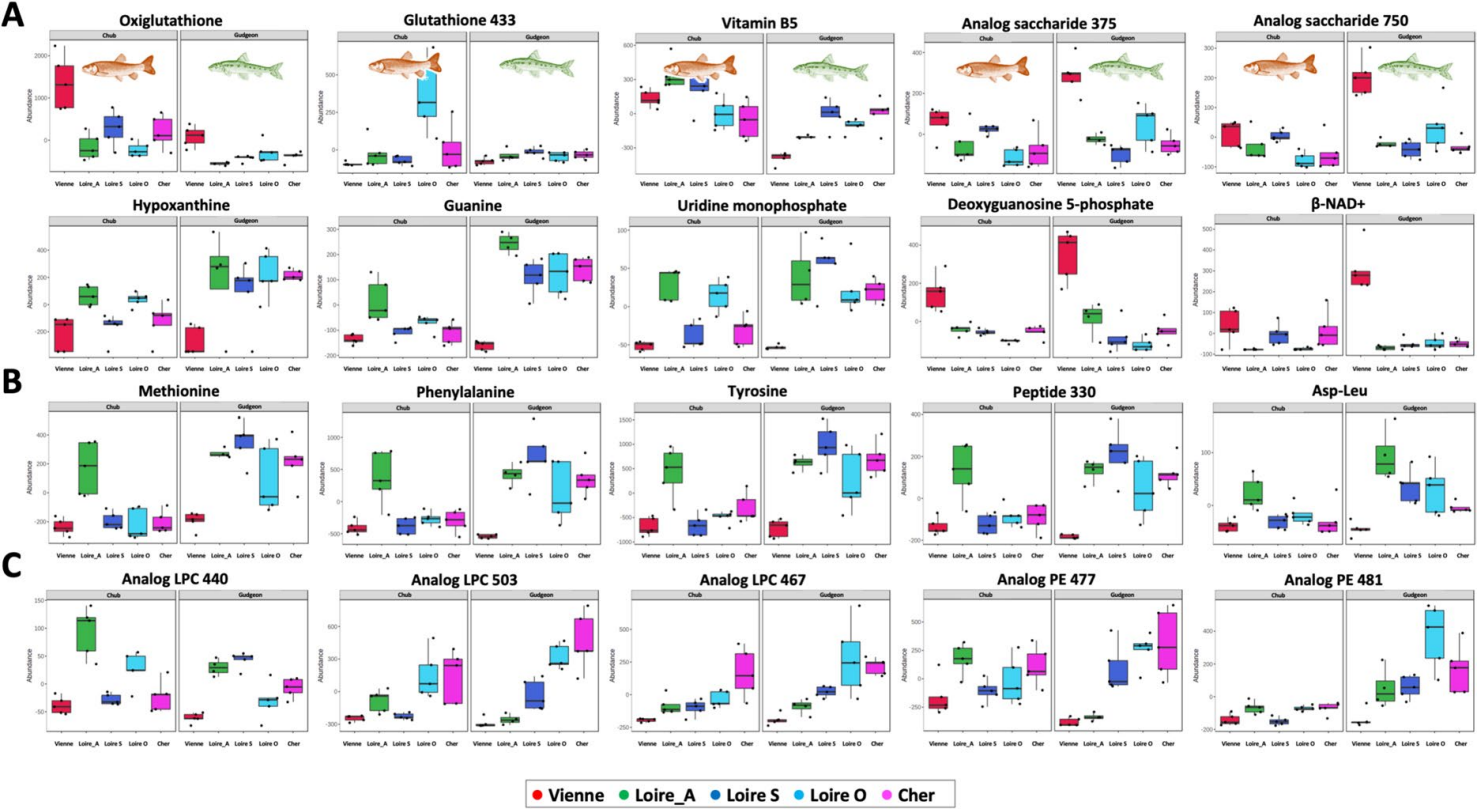
Boxplots of annotated metabolites presenting significant abundance differences showing the effect of the sampling location on chub and/or gudgeon muscle metabolomes. Plots show different content in metabolites related to oxidative stress and energy catabolism (**A**), amino acids (**B**), and lipids (**C**), amino acids especially when comparing the Vienne samples to others (ANOVA 2; *p* < 0.001; *n* = 49)

Boxplots of selected annotated metabolites illustrate these differences (Fig. 4; Supplementary Figures S4–S5). In the gut, chubs and gudgeons from Vienne had elevated levels of oxidized glutathione, amino acids, and lipids and decreased nucleic acids (e.g., guanine and guanosine). The liver showed similar trends: increased oxidized glutathione and lactones, with reductions in nucleic acids (e.g., xanthine and hypoxanthine), peptides, and lipids. In muscle, higher levels of oxidized glutathione, deoxyguanosine 5-phosphate, and NAD⁺ were observed, alongside reductions in vitamin B5, reduced glutathione, various nucleic acids, amino acids, and membrane lipids (LPCs and PEs).

Environmental analysis of the benthic biofilms revealed high densities of Oscillatoriales cyanobacteria (Phormidiaceae, Nostocales, Oscillatoriaceae) and elevated levels of anatoxin-a in Vienne samples collected on July 24, 2019 (Supplementary Figure S6), supporting the hypothesis of localized environmental stress

## Discussion

### Fish-based environmental metabolomics

Comparative environmental metabolomics applied to fish across stress gradients offers valuable insights into their biology and ecology (Pomfret et al., 2020; Walker et al. 2022; Oliveira et al. 2024). This form of chemical phenotyping can uncover physiological, ecological, and even evolutionary processes, providing a basis for developing bioindication strategies (Izral et al. 2021; Chen et al. 2021; De Marco et al. 2021; Sparagon et al. 2022; Long et al. 2020). The effectiveness and reliability of bio-indicative markers, as revealed through metabolomics, depend on rigorous validation and well-designed experimental frameworks (Schütz et al. 2021; Legrand et al. 2023).

The use of LC–MS-based metabolomics has recently gained prominence for assessing the impacts and modes of action of environmental stressors and contaminants (Beyoğlu and Idle 2020). While widely used in ecotoxicological studies under controlled laboratory conditions (Le Manach et al. 2018; Ekman et al. 2015; Gil-Solsona et al. 2017), its application in environmental field research remains comparatively limited (Bundy et al. 2009; Lankadurai et al. 2013) until recently. Nonetheless, alongside several NMR-based studies (Capello et al., 2016; Sotton et al. 2019; Wei et al. 2018), a growing number of successful field applications demonstrate the potential of LC–MS metabolomics to enhance our understanding of in situ biological, physiological, ecological, and ecotoxicological dynamics (Meador et al. 2020; Reverter et al. 2017; Goode et al. 2020).

### Potential of high-throughput metabolomics for environmental assessment

Our study represents one of the early efforts to explore the potential of high-throughput molecular phenotyping—specifically LC–MS-based metabolomics—for environmental assessment. This organism-centered approach, combined with multivariate chemometric analysis, yields valuable insights into phenotypic plasticity and biological responses to environmental conditions. By linking molecular variability and responsiveness to local environmental factors, it becomes possible to identify correlations—and potentially causal relationships—between stressors and biological effects. In this case, the most pronounced metabolomic shifts, including consistent stress-related molecular signatures in both fish species, were observed at the Vienne River site and coincide with the presence of anatoxin-a-producing cyanobacterial biofilms.

Although our findings do not provide direct evidence of anatoxin-a impact on chubs and gudgeons from the Vienne River, several observations suggest that anatoxin-a production by cyanobacterial biofilms may pose a significant threat to fish in colonized rivers. Indeed, remarkably high or low content of certain key metabolites was specifically noticed in the different tissues of fish caught from the river Vienne, which may traduce ecotoxicological and/or physiological impairments potentially induced by anatoxin-a. One striking example concerns glutathione content, as higher oxiglutathione associated with lower reduced glutathione observed in tissues of fish from the river Vienne constitutes noticeable stress-related markers (Asensi et al. 1999). Also, lower nucleic acid (xanthine, guanine, hypoxanthine, guanosine, or UdP) and amino acid (valine, tryptophan, tyrosine, or methionine), small peptide (Glu-Phe or Asp-Leu), or lipophosphocholine (LPC) contents might be related to higher consumption of general metabolism reserves (Collet et al. 2017). On the contrary, the higher dG5P and NAD +, together with saccharide contents, may rather specifically reflect higher oxidative capabilities in the muscle metabolomes of fishes from the river Vienne (Peek et al. 2013).

In a recent controlled experiment on Medaka fish, a 4-day exposure to environmentally relevant concentrations of various *Microcystis* strains induced marked metabolomic alterations, depending on the bioactive metabolite composition of each strain (Marie et al. 2025). Notably, gut and muscle metabolomes displayed stronger interspecific differences between chubs and gudgeons than the liver, highlighting organ-specific stress responsiveness that may be shaped by the specific cyanobacterial metabolites present.

Our previous environmental study on fish from lakes with varying cyanobacterial communities showed that liver metabolomes could be linked to the presence of potentially toxic species (Sotton et al. 2019; Marie and Gallet 2022). Additionally, in earlier experiments, we observed that Medaka fish exposed to sub-lethal doses of anatoxin-a exhibited a rapid detoxification response, with the toxin becoming nearly undetectable 12 h post-exposure. Despite this rapid clearance, the liver metabolome revealed a clear stress response, including changes in oxidized glutathione and purine derivatives—markers indicative of cellular disruption (Colas et al. 2020; 2021).

While we could not detect anatoxin-a in fish tissue from the Vienne River—possibly due to limitations in detection sensitivity or efficient detoxification during chronic exposure—our results highlight the need for further investigation into the potential sub-lethal effects of anatoxin-a-producing cyanobacterial mats on fish metabolomes.

### Gut, liver, and muscle informativeness and specificities

Environmental metabolomics, when applied to organ-specific analyses across multiple fish species, offers a unique lens through which to examine the complex interplay between environmental conditions, organismal physiology, and adaptive responses (Bodin et al. 2022). A key and unexpected finding of this study is that fish muscle exhibits more pronounced metabolomic differences in response to local environmental variation than either the gut or liver. This observation opens new avenues for the operational use of muscle metabolomics in ecological assessments, potentially extending the concept of “terroir” to natural ecosystems (Ducarme 2025).

The gut serves both as a barrier and an entry point for environmental contaminants and hosts a diverse microbiota that plays a critical role in digestion, homeostasis, and immunity (Xiong et al. 2019). As the first physiological interface between fish and their environment, the gut is central to maintaining internal balance and responding to external stressors (Gallet et al. 2023). In contrast, the liver is a key metabolic organ involved in digestion, hormone synthesis, and detoxification. It exhibits strong organotropism for xenobiotics and carries out biotransformation through phase I and II enzymatic processes (Matsuo et al. 2008; Sabir et al. 2019). Due to its central role in managing physiological and chemical stress, the liver metabolome has been widely studied in ecotoxicological research (Capello et al. 2016; Sotton et al. 2019; Gil-Solsona et al. 2017; Beyoğlu and Idle 2020).

By comparison, muscle is traditionally seen as a specialized tissue for locomotion and has received less attention in environmental studies, despite its important functional and biochemical roles (Bodin et al. 2022). It serves as a major reservoir for key metabolites such as lipids, amino acids, nucleotides, and sugars—many of which contribute to the gustative qualities associated with terroir in edible fish (Muroya et al. 2020). Notably, in our study, muscle metabolomes exhibited the most distinct spatial variation across sampling sites, independent of species. This suggests that muscle may be a particularly sensitive and underutilized bioindicator, potentially more reflective of local environmental influences than the gut or liver.

### Molecular next-generation bio-indicators?

Fish have long served as reference models in aquatic ecology to assess physical and chemical stressors, using a range of traits such as behavior (Almroth et al. 2021) and stress-related enzyme activity (Villeneuve et al. 2021), alongside traditional ecological indicators (Harris 1995; Benejam et al. 2008). However, no single trait has consistently provided a reliable bio-indicative signal. Emerging omics approaches now offer the potential for more integrative, multidimensional signatures that better capture organism–environment interactions (Marie 2020). To this end, specific reflection regarding sampling design has been specifically tested, now claiming the consideration of a large set of environments comprising several potential “control” sites that would serve as multiple references compared to several attemptedly stressed locations to highlight contrasted bioindicative signatures (Simmons et al. 2015).

Among these, the metabolome has emerged as a particularly promising multifunctional molecular trait for environmental and ecotoxicological studies (Martynuik and Simmons 2016). Metabolomics offers key advantages, including its close linkage to the organism’s overall physiological state (Rosato et al. 2018), and its applicability across both model and non-model sentinel species such as fish (Hillyer et al. 2021; Reverter et al. 2017).

To address ethical concerns in bio-monitoring, there is growing interest in developing less invasive or non-lethal sampling techniques. Recent advances in LC-HRMS have enabled sensitive metabolomic analysis from minimal biological material, opening the door to non-lethal bioindicators such as gill mucus collected via swabs, blood from the caudal vein, or even fecal samples. However, the bio-indicative value of these alternative matrices—and the potential physiological impact of collection methods, including cold, chemical, or electric anesthesia—remains to be systematically evaluated (Colette et al. 2016; Reverter et al. 2017; Ivanova et al. 2021; Sylvain et al. 2023).

## Conclusions

When it comes to emerging biomarkers for diagnosing fish health, there is a need to improve diagnostic tools. This can be achieved by developing non-invasive methods and identifying biomarkers for the early detection of environmental changes. However, the application of metabolomics to environmental issues is still limited by a lack of conceptual, technical, and data frameworks that need to be customized to fully realize its potential for practical environmental purposes (Walker et al. 2022).

## Supplementary Information

Below is the link to the electronic supplementary material.

ESM1 (XLSX 3.17 MB)

ESM2 (DOCX 7.65 MB)

ESM3 (PDF 1.17 MB)

## Data Availability

Data is provided within the manuscript or supplementary information files.
