# Supplementary material for: Metabolomic fingerprinting of soft tissues uncovers taxonomic, physiological, and ecological aspects of river fishes
Source: Fish Physiol Biochem. 2026 Jan 22;52(1):18. doi: 10.1007/s10695-026-01638-8 (PMC12827312; doi:10.1007/s10695-026-01638-8)
Supplement: Supplementary file 2 — (DOCX 7.65 MB) [file 10695_2026_1638_MOESM2_ESM.docx]

**Supplementary figures**

**Supplementary figure S1.** Results of the two-ways ANOVA analyses performed on the metabolome dataset of the guts (A), livers (B) and muscles (C) of the fish of 49 chubs and gudgeons collected in the Vienne, Cher and Loire rivers during the 2019’s, considering the species and the sampling location factor and their interaction.

**Supplementary figure S2.** Molecular networks generated from 895 MS/MS spectra obtained from the 3 tissues (gut, liver and muscle) of chubs and gudgeons (n=49) using the GNPS. The graph displays connected metabolites sharing a structural similarity based on the similarity of their respective fragmentation patterns. Homolog and analog annotated compounds are indicated in red and green colours, respectively.

**Supplementary figure S3.** Individual plot of PLS-DA performed with guts (A and D), livers (B and E) and muscles (C and F) of the fish of 49 chubs and gudgeons considering their respective species (A-C) and their sampling location (D-F).

**Supplementary figure S4.** Boxplots of boxplots of annotated metabolites presenting significant abundance differences regarding the impact of the sampling location on chub and/or gudgeon gut metabolomes show higher (A) or lower (B) contents in Vienne’s samples (ANOVA 2; p<0.001).

**Supplementary figure S5.** Boxplots of boxplots of annotated metabolites presenting significant abundance differences regarding the impact of the sampling location on chub and/or gudgeon liver metabolomes show higher (A) or lower (B) contents in Vienne’s samples (ANOVA 2; p<0.001).

**Supplementary figure S6.** Bacterial composition of the biofilms collected in the same area fish sampling regarding in-depth sequencing of 16S rRNA encoding *V4-V5* fragment (Duperron et al., 2023) (A) and corresponding anatoxin-a contents measured by targeted LC-HRMS (colas et al., 2020) (B) suggest that Oscillatoriaceae observed in the Vienne’s biofilm may constitute high anatoxin-a producers. Results correspond to media of triplicated analyses.
