# Supplementary figures and images for: Metabolomic fingerprinting of soft tissues uncovers taxonomic, physiological, and ecological aspects of river fishes
Source: Fish Physiol Biochem. 2026 Jan 22;52(1):18. doi: 10.1007/s10695-026-01638-8 (PMC12827312; doi:10.1007/s10695-026-01638-8)

### Metabolome Vs species

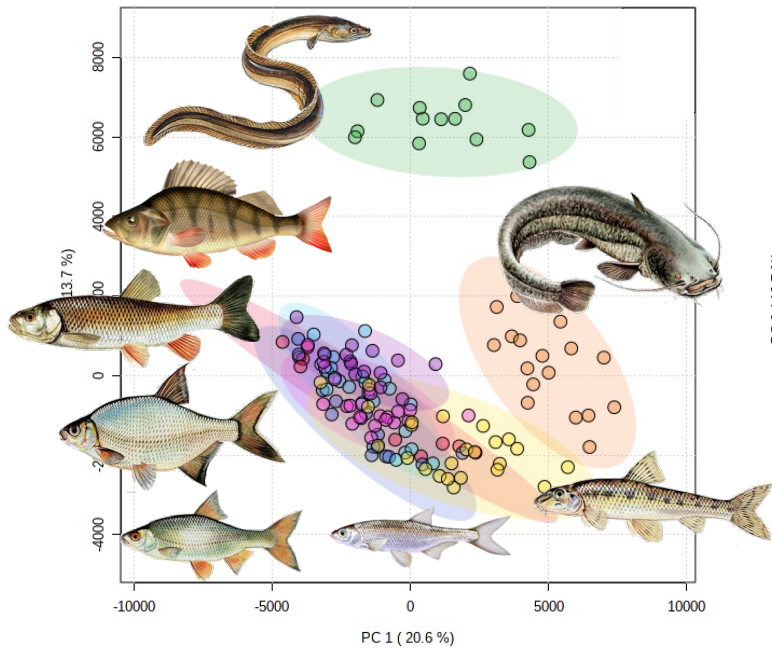

### Metabolome Vs organs

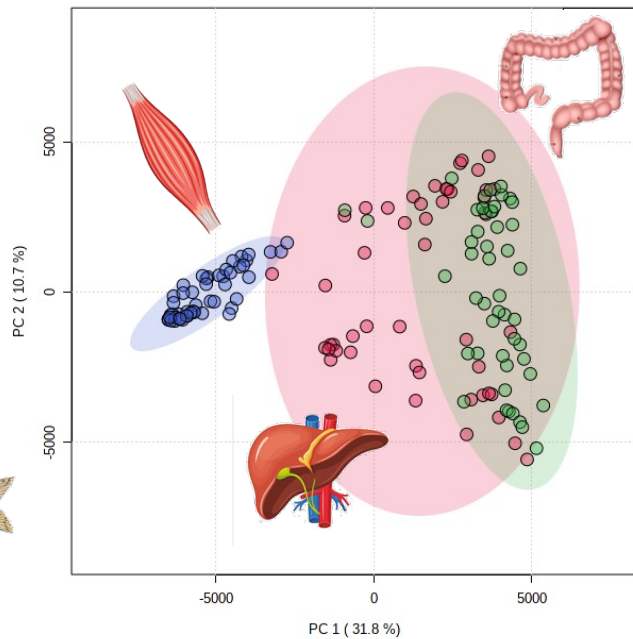

### Metabolome Vs sampling

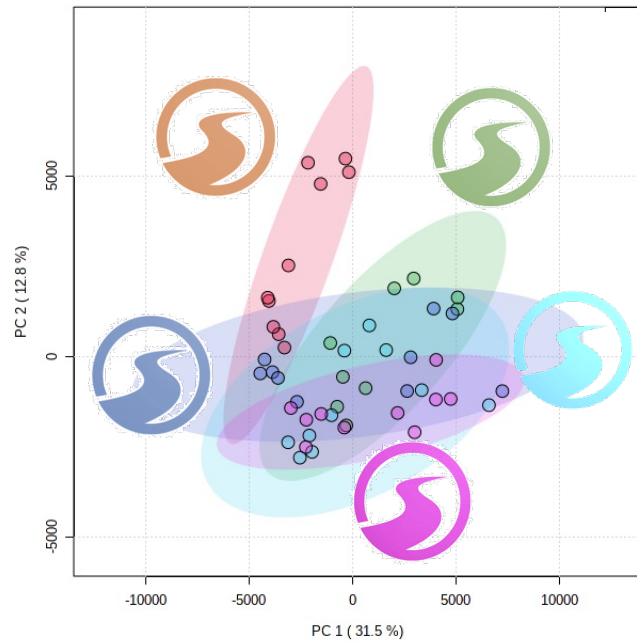

Supplement: Supplementary file 3 — (PDF 1.17 MB) [file 10695_2026_1638_MOESM3_ESM.pdf]
